# Blood-brain barrier integrity of stroke patients presenting in an extended time window

**DOI:** 10.1186/s12883-020-01634-2

**Published:** 2020-02-13

**Authors:** Jarrhett Butler, Parisa Heidari, Sarah Blayney, Emi Hitomi, Marie Luby, Richard Leigh

**Affiliations:** National Institute of Neurological Disorders and Stroke, National Institutes of Health, Building 10, B1D733 MSC 1063, 10 Center Drive, Bethesda, MD 20892 USA

**Keywords:** Stroke, Thrombolysis, Blood-brain barrier, Permeability imaging, Extended time window

## Abstract

**Background:**

Current guidelines limit thrombolytic treatment of stroke to those patients who present within 4.5 h to minimize the risk of hemorrhagic complications. Risk of hemorrhage increases with increasing blood-brain barrier (BBB) disruption. This study aimed to determine, in a cohort of patients presenting outside of an IV-tPA treatment window, whether disruption of the BBB is time dependent, and what proportion of patients could be safely treated.

**Methods:**

We analyzed untreated stroke patients, seen between 2011 and 2015, who had MRI studies in the time window of 4 to 24 h from symptoms onset. Permeability of the BBB was measured within the ischemic tissue using an application of dynamic susceptibility contrast imaging. Patients were dichotomized into two groups based on a 20% threshold of BBB disruption and compared using logistic regression.

**Results:**

Of the 222 patients included in the final analysis, over half, 129 (58%), had preserved BBB integrity below the 20% threshold. There was no relationship between time imaged after symptom onset and the amount of BBB disruption (*p* = 0.138) across the population; BBB disruption varied widely.

**Conclusions:**

Estimating BBB integrity may help to expand the treatment window for stroke patients by identifying those individuals for whom thrombolytic therapy can be considered.

## Background

The use of intravenous tissue plasminogen activator (IV-tPA) was initially approved for treatment of acute ischemic stroke (AIS) when administered within 3 h of stroke onset [1] and subsequently was adopted for treatment out to 4.5 h from onset [2]. For patients presenting greater than 4.5 h from onset, there are currently no approved thrombolytic treatment options according to current guidelines [3]. Presenting to the hospital beyond this time window is the main reason an AIS patient is not treated with IV-tPA [4]. The reason for withholding IV-tPA in the extended time window is in part due to the concern for an increased risk of hemorrhagic transformation (HT) which may negate any potential benefits of the drug [5]. Specifically the development of a space occupying parenchymal hematoma exerting mass effect, often referred to as a PH-2 [6], can be associated with clinical deterioration [7].

AIS is known to affect the integrity of the blood-brain barrier (BBB). While mild BBB disruption is reversible with reperfusion, severe focal BBB disruption is associated with HT [8]. There is a dose dependency between the severity of BBB disruption within the ischemic lesion and the severity of the HT in patients treated with IV-tPA [9] or endovascular therapy [10], a relationship that has been confirmed using multiple BBB-imaging approaches [11, 12]. However, imaging of the BBB has not previously been used for prospective selection of patients in clinical trials of thrombolysis in the extended time window (beyond 4.5 h).

Recent studies have found that patients presenting in extended time windows do have salvageable tissue on multimodal imaging that benefits from thrombolysis [13–15]. Given the need to expand the number of AIS patients who can safely be treated with IV-tPA, identification of potential patients who present in an extended time window by measuring BBB integrity offers an opportunity to improve outcome. The purpose of this study was to measure BBB integrity in a cohort of patients presenting in an extended time window to determine if BBB disruption worsens with time in a consistent manner and to estimate the proportion of patients who potentially may be safe to treat with thrombolysis based on preserved BBB integrity.

## Methods

### Patient cohort

This research was conducted as a retrospective analysis of de-identified registry data, for which we obtained a determination of *Not Human Subjects Research* from the NIH Office of Human Subjects Research Protections (OHSRP).

Patients seen by the NIH stroke team at two area hospitals (MedStar Washington Hospital Center and Suburban Hospital) during the 5-year period from the beginning of 2011 to the end of 2015 were included in the study if they met the following criteria: 1) diagnosis of acute stroke or TIA; 2) assessment by stroke team > 4 h but < 24 h from last seen normal; 3) did not receive any acute treatment. Patients were excluded if: 1) no MRI scan was performed in the 4–24 h time window; 2) no perfusion weighted imaging (PWI) was available for the BBB analysis; 3) the absence of ongoing ischemia at the time of MRI defined as a lesion on PWI. Lesions on PWI were defined by a threshold of 4 s in delay of contrast delivery relative to the contralateral hemisphere.

### MRI protocol

Images were acquired on a 1.5 T GE Signa scanner (General Electric Medical Systems, Milwaukee, WI), a 3 T Philips Achieva scanner (Philips Healthcare, Best, The Netherlands), or a 3 T Siemens Skyra scanner (Siemens AG, Munich, Germany). Image sequences and typical parameter ranges were: diffusion tensor imaging (TR 4461–10,500 msec, TE 61.6–92.8 msec, 3.5 mm slice thickness, 40 slices) used to generate trace diffusion weighted images (DWI) using three orthogonal directions (b = 0 and 1000 s/mm^2^) and apparent diffusion coefficient (ADC) maps; fluid attenuated inversion recovery (FLAIR) imaging (TR 9000–9002 msec, TE 120–147 msec, 3.5 mm slice thickness, 40 slices); time-of-flight (TOF) magnetic resonance angiography (MRA) images (TR 18–23 msec, TE 3.43–6.8 msec, 0.75–289.3 mm slice thickness, 73–95 slices); gradient recall echo (GRE) images (TR 700–800 msec, TE 12–20.55 msec, 3.5–7 mm slice thickness, 20–40 slices); dynamic susceptibility contrast (DSC) perfusion weighted imaging (PWI) (TR 1–1.5 s, TE 25–45 msec, 7 mm slice thickness, 20 slices, 40–80 dynamics), which was collected during a single injection of a weight-based dose of gadolinium (0.1 mmol/kg of gadolinium-DPTA, Magnevist; Bayer Schering Pharma, Whippany, New Jersey or gadolinium-BOPTA Multihance, Bracco Diagnostics, Monroe Township, New Jersey) at a flow rate of 5 mL/sec. Although MRI vendor, strength, and parameters varied between sites and over time, every attempt was made to ensure the resulting images were similar in their properties.

### Blood-brain permeability imaging (BBPI) analysis

The method for calculating mean permeability derangement (MPD) was the same as was used and described in two prior studies [9, 10]. Blood-brain permeability imaging (BBPI) is a method for calculating BBB permeability from the source images of a dynamic susceptibility contrast (DSC) image acquisition. DSC imaging is collected for clinical purposes to generate PWI maps, however the source images can also be post-processed to create BBB permeability images as was done in this study. DSC collects T2*-weighted images of the brain at a frequency of one volume every 1–1.5 seconds just prior to, during, and after a weight-based dose of gadolinium is administered with a power injector. On a DSC image, intravascular gadolinium causes a T2* susceptibility artifact that allows for bolus tracking and the generation of various PWI maps such as time-to-peak (TTP) concentration. However, when gadolinium leaks through the BBB and into tissue parenchyma, the recorded signal also has a T1 component that is proportional to the concentration of gadolinium in the tissue voxel [16]. In the setting of BBB disruption, the gadolinium concentration curve is shifted down resulting in an under-estimation of the cerebral blood volume [17]. The amount of change in signal caused by the leakage of gadolinium can be quantified relative to normal tissue as a value referred to as K2. However in the setting of a perfusion deficit, an arrival time correction must first be applied [18]. BBPI generates K2 values for each voxel in the brain after applying the arrival time correction. The resulting K2 value is a number between zero and one that can be expressed as an index or a percent. This number reflects the percentage change in the recorded signal due to the effect of BBB disruption and is a relative number that is unitless.

BBPI was generated for all patients in this study using the contralateral hemisphere as the normal reference. Regions of interest (ROI) were created in the affected hemisphere based on a threshold of 4 s delay relative to normal on the TTP map. Relative delay in TTP has been found to be equivalent to other methods of identifying ischemia but does not require deconvolution of an arterial input function (AIF) making it less susceptible to errors introduced by AIF selection [19, 20]. The ROIs were then placed on the BBPIs and mean permeability derangement (MPD) was calculated. MPD is defined as the mean K2 value of all voxels in the ROI that are two standard deviations above normal. This approach has been used to identify patients with areas of focal BBB disruption. Stroke volumes were calculated by placing the co-registered ROI from the PWI-TTP on the apparent diffusion coefficient (ADC) map from the diffusion weighted imaging (DWI). The DWI volume was defined by the voxels within the ROI with an ADC value < 620 μm/sec. Image analysis was performed in Matlab (Mathworks, Natick, MA).

Prior studies have identified a MPD cut off of 20% beyond which the risk of developing a PH-2 hemorrhagic transformation increases significantly [9, 10]. Thus we dichotomized patients into two groups, those with preserved BBB integrity defined as MPD < 20% and those with BBB rupture defined as MPD > 20%. Variables tested included age, sex, stroke severity (NIHSS), volume of perfusion lesion, volume of diffusion restriction, and time to MRI. Time to MRI was examined based on three variables: time from last seen normal (LSN) to MRI, time from symptom discovery (SD) to MRI, and approximate time of onset to MRI. Approximate time of onset was calculated from the midpoint between LSN and SD, a measure that has been used in other studies of patients presenting in an extended time window [14, 15].

### Statistical analysis

Preserved BBB integrity was treated as a binary outcome and compared with clinical and radiographic features using logistic regression. A multivariate analysis was performed on variables with a *p*-value < 0.1; a *p*-value of < 0.05 was considered significant. Statistical analysis was performed in STATA 13 (StataCorp LLC, College Station, TX).

## Results

**Fig. 1:**
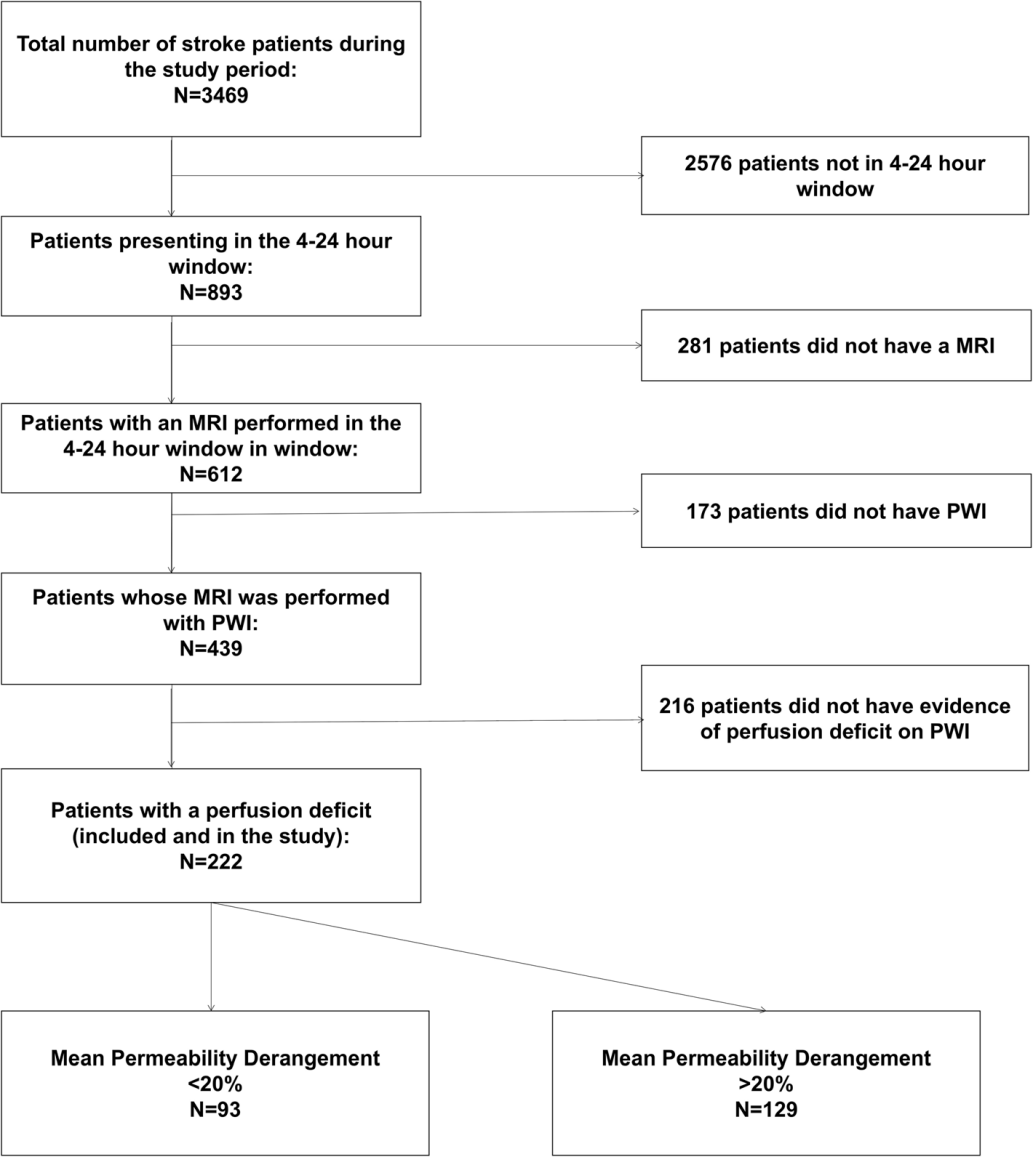
A flow chart details how the population included in the study was determined

Of the 3469 patients with stroke or TIA seen by our stroke service during the study period, 893 presented with a known time of LSN that was in the 4–24-h window, of whom 612 had an MRI. PWI was performed on 439 of these patients; 222 had a quantifiable PWI lesion on their TTP map and were included in the analysis (Fig. 1). Baseline characteristics of the patient population are shown in Table 1. Median age was 73, and 55% were women. The median NIHSS was 6. The median size of the ischemic lesion on PWI was 18.5 mL, and the median size of the stroke on DWI was 2.95 mL. Mean time from LSN to MRI was 677 min (or 11.3 h), while the average time from SD to MRI was 367 min (or 6.1 h). Approximate onset (midpoint between LSM and SD) to MRI was 511 min (or 8.5 h).

**Table 1:** Continuous variables are shown as mean ± SD, NIHSS and Age are shown as median and IQR, and categorical variables are shown as total (percent). DWI indicated diffusion-weighted imaging; NIHSS, National Institute of Health Stroke Scale; PWI, Perfusion-weighted Imaging; tPA, tissue-type plasminogen activator

	All Patients (*N* = 222)	< 20% Permeability (*N* = 129)	> 20% Permeability (*N* = 93)	Univariate Analysis	Multivariate Analysis
Mean Permeability Derangement (%)	27.97	12.45	49.45	–	
Age (median)	73 (59–85)	69 (58–83)	76(64–86)	*p* = 0.031	*p* = 0.019
Sex (%)	54.5	51.9	57.4	*p* = 0.238	
NIHSS (median)	6 (0–14)	6 (3–13)	6(2–14)	*p* = 0.695	
PWI Volume (mL)	18.5 (4.3–69.56)	26.43 (8.9–83.72)	10.42 (2.7–41.55)	p = 0.012	*p* = 0.007
DWI Volume (mL)	2.95 (0.5–9.13)	5.1 (0.82–11.19)	1.132 (0.3383–6.86)	*p* = 0.24	
Last Seen Normal to MRI (minutes)	677 ± 303.5	681 ± 300.32	670 ± 309.50	*p* = 0.781	
Found with Symptoms to MRI (minutes)	364 ± 302.65	389 ± 330.05	327 ± 356.10	*p* = 0.138	
Approximate Onset to MRI (minutes)	511 ± 275.6	531 ± 287.50	482 ±257	*p* = 0.195	

**Fig. 2:**
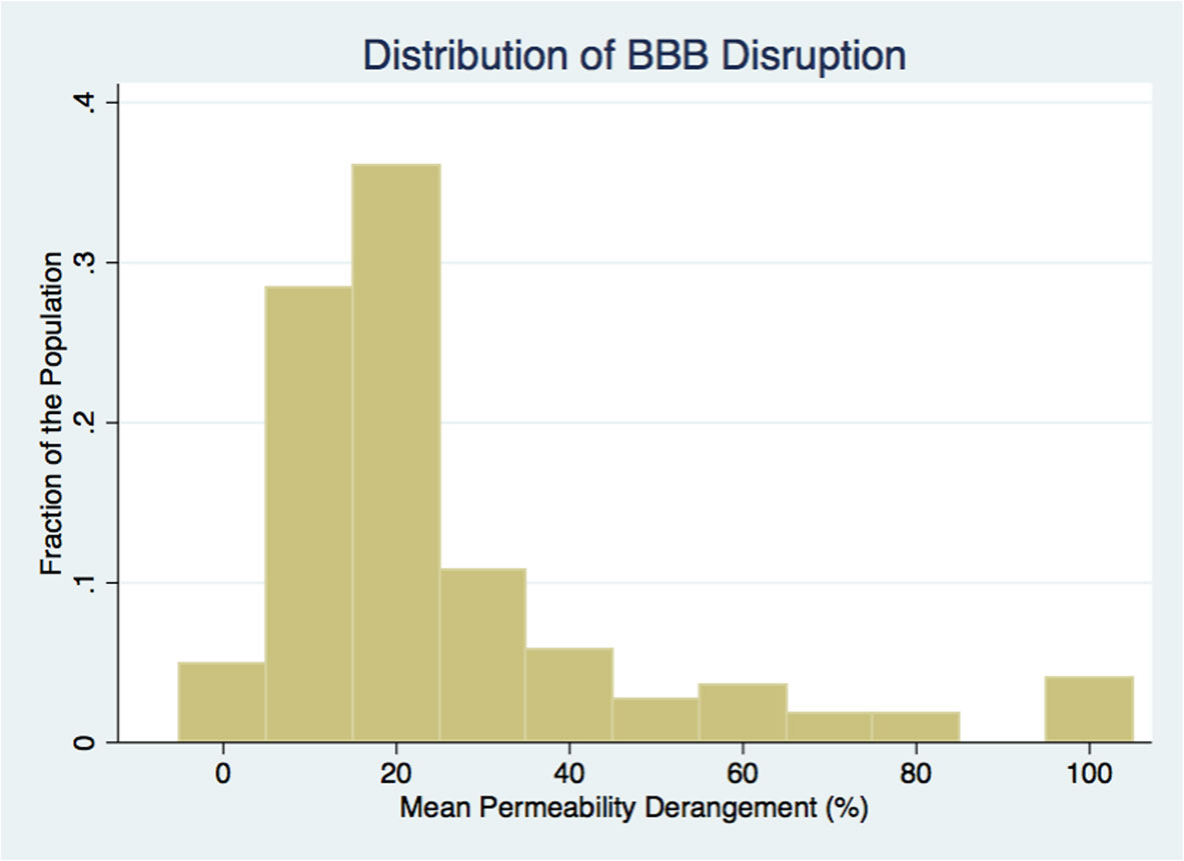
A histogram shows how the mean permeability derangement was distributed across the population

**Fig. 3:**
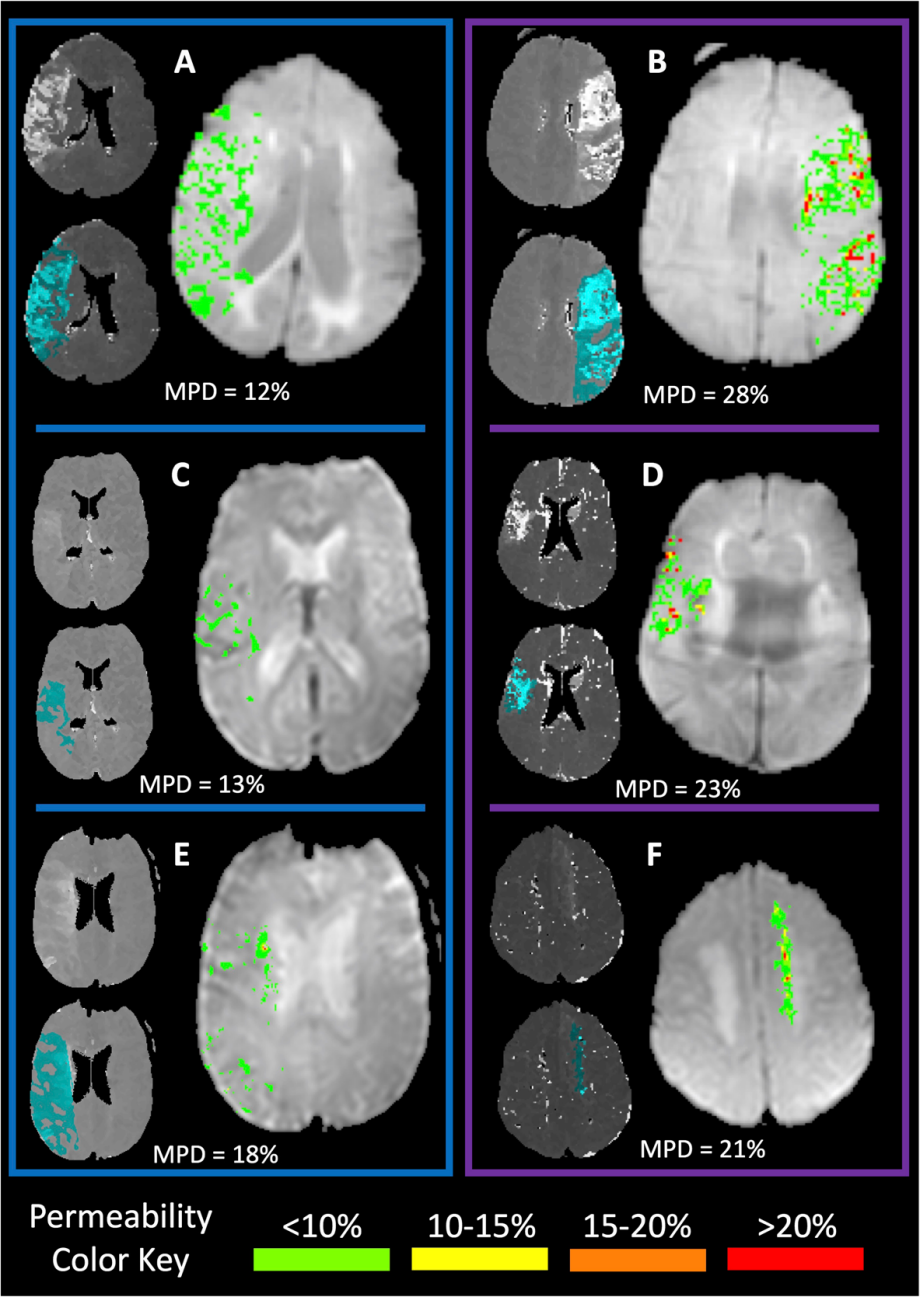
Permeability imaging from six different patients is shown. Each panel has two thumbnail images and a larger permeability map. In each case, the upper thumbnail image is the time-to-peak (TTP) map and the lower thumbnail is the TTP map with the region of ischemia (relative TTP > 4 s) shaded in light blue. To the right of the thumbnails is the blood-brain permeability heat map within the regions of ischemia, color coded according to the color key at the bottom of the figure. The mean permeability derangement (MPD) is indicated in each panel for each patient. The three panels in the blue box on the left have MPD < 20%, whereas those in the purple box on the right have MPD > 20%. Panels **a** and **b** contrast two patients with large perfusion deficits, one below the threshold and one above. Similarly, panels **c** and **d** contrast two patients with more distal vessel occlusions. Panels E and F show two patients with an MPD very close to the threshold

The average MPD for the entire cohort was 28%. Figure 2 shows a histogram of how MPD was distributed across the population. Using an MPD threshold of 20% to dichotomize patients, 129/222 (58%) had an MPD consistent with preserved BBB integrity and may have been safely treated with IV-tPA. Conversely, had the entire population been treated with IV-tPA, 42% potentially would have sustained a severe hemorrhagic complication (PH-2). The characteristics of the two groups are shown in Table 1. Figure 3 shows the BBB heat maps for six patients, three with an MPD below the 20% threshold and three with an MPD above the threshold.

**Fig. 4:**
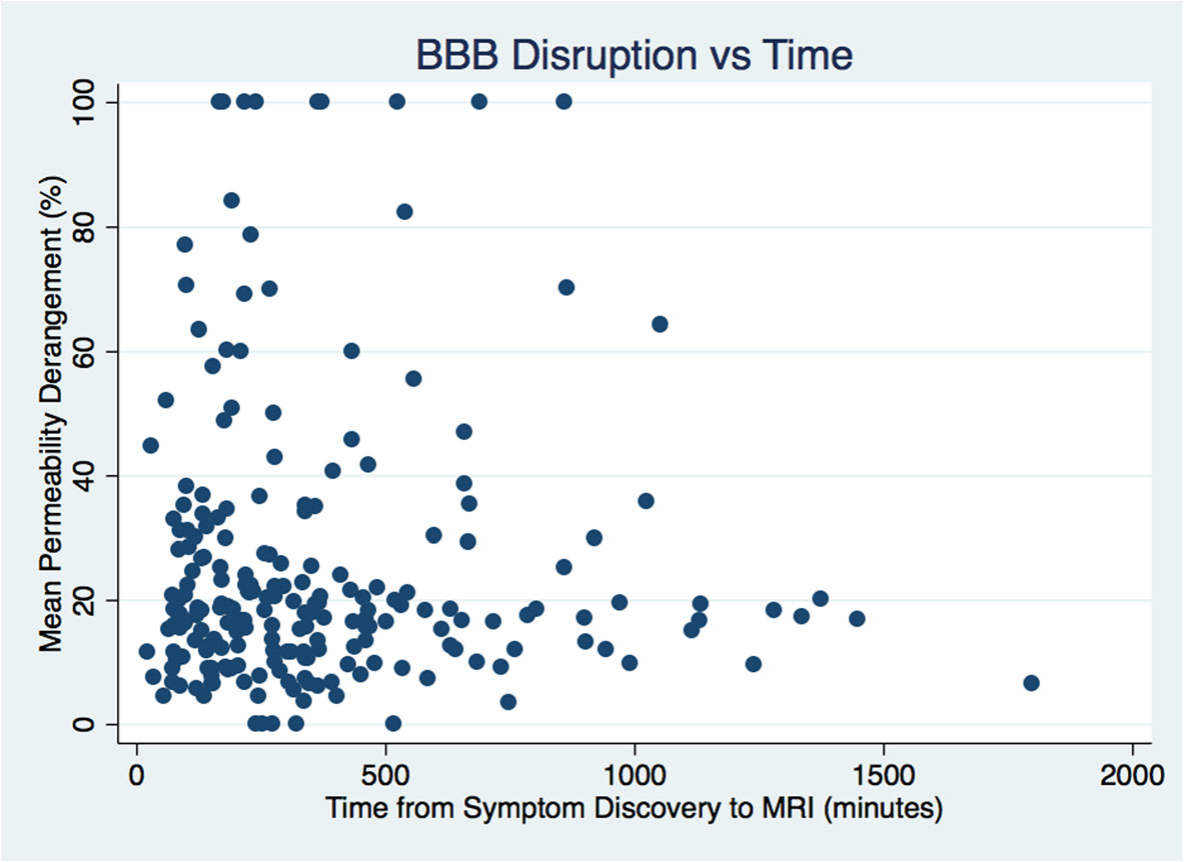
A scatter plot compares mean permeability derangement with time from symptom discovery

In the univariate analysis, the group with preserved BBB integrity was significantly younger (OR 1.02, CI 1.002:1.036, *p* = 0.031) but also had significantly larger PWI deficits (OR 0.994, CI 0.989:0.999, *p* = 0.012). These variables remained independently associated with BBB integrity in the multivariate analysis. There was no sex difference detected. None of the three time-metrics showed a significant relationship to BBB integrity: LSN to MRI (*p* = 0.781), SD to MRI (*p* = 0.138) and approximate onset to MRI (*p* = 0.195). Figure 4 shows a scatter plot of time from SD to MRI compared with the MPD.

Applying a stricter MPD threshold of 10% identified 43/222 (19%) patients who would potentially be very safe to treat with thrombolysis. Thus, even when taking a more cautious approach to treatment, 1 in 5 patients presenting in an extended time window may be safe to treat. Looking at only those explicitly known to be in an extended time window (having an interval between SD and time of MRI that was greater than 4.5 h), 72/111 (65%) had an MPD below the 20% threshold and 22/111 (20%) had an MPD below the 10% threshold. These numbers were similar to those of the entire cohort, suggesting that unknown onset time does not affect the findings.

## Discussion

This study has two key findings: 1) the distribution of BBB disruption within ischemic tissue varied widely within the population of patients studied; 2) at the population level, there was no clear dependence between the time from symptom onset to imaging and the severity of BBB disruption. This does not exclude the possibility that there is a time dependence for BBB disruption within a patient; rather it suggests that each patient may progress at their own rate. A subset of the population was found to have preserved BBB integrity several hours beyond the current guidelines for treating with IV-tPA. These findings suggest there is an opportunity to treat many more patients than are currently being treated. However, there are also several caveats to these findings. The threshold used, while based on previous work, has not been validated and may not be correct. There may be factors that contribute to the risk of HT in addition to BBB disruption that are not captured in this study. And lastly, treating patients in an extended time window would only be expected to be beneficial if the underlying tissue was salvageable.

Although this study is not able to answer the question of benefit, other studies have found that multimodal imaging can identify patients who benefit from reperfusion in an extended time window [21, 22]. Although initial studies were limited to patients with large vessel occlusion who can be treated with mechanical thrombectomy, more recent trials using multimodal imaging to treat with IV-tPA have also shown benefit [13, 14]. Thus, imaging the integrity of the BBB may add to these findings by improving the safety of this practice. BBB imaging may also be helpful in other situations where IV-tPA is currently withheld, such as in patients taking oral anticoagulants.

It is also important to distinguish between patients who are in an unknown time window from those who are explicitly in an extended time window. Patients who have an unknown time of onset but are FLAIR negative can be safely and effectively treated with IV-tPA [23, 24]. In this special case, it is assumed the patient is actually in an early time window due to the known temporal changes of FLAIR signal within an acute infarct. In both trials testing FLAIR-guided thrombolysis, patients known to be in an extended time window were excluded. Since waking with stroke symptoms is a relatively common presenting scenario, wake-up stroke has typically constituted half of the patients enrolled in trials testing treatment in an extended time window [13, 21, 22]. In the current study, patients who were explicitly known to be in an extended time window had a similar distribution of BBB integrity as the entire population, again suggesting that time is not a surrogate for assessing the risk of HT. Thus, these results may help identify wake-up stroke patients who are FLAIR positive who would be safe to treat. However, further investigations are needed to assess the relationship between FLAIR change and BBB disruption.

One question that is raised by this study is: why is there so much variability in BBB disruption across the cohort? And if time is not the central factor, what is? Presumably the reason a patient with severe BBB disruption is at higher risk for hemorrhagic complications is that BBB disruption is serving as a biomarker for the severity of the ischemic injury. Thus, while mild BBB disruption reflects dysfunction of the BBB, severe BBB disruption represents BBB rupture. Factors like collateralization or genetic predisposition may play a role. We found that older age, which is known to be associated with increased BBB permeability in general [25], was associated with poorer BBB integrity. This heterogeneity supports the movement toward more personalized medicine and the use of multimodal imaging to make more individualized clinical management decisions. We also found that patients with larger perfusion deficits had less BBB disruption despite there being no significant difference in the size of the strokes on DWI. This may be due to patients in our cohort with larger deficits having better collateral circulation preventing infarct growth. It is known that penumbral imaging can identify patients who benefit from treatment in an extended time window [3], presumably due to robust collateralization. It may be that collateralization is also protecting the BBB. Future studies will explore this relationship. It is also possible that some patients may have had partial recanalization of their initial perfusion deficit at these late time points which could lead to smaller PWI lesions that had more substantial BBB disruption due to post-reperfusion (biphasic) opening of the BBB [26]. Additionally, comorbid disease states such as hyperglycemia and diabetes, which were not collected as part of this dataset, are known to affect the BBB [9] and may have contributed to the variability.

When reviewing the various clinical trials testing the safety of IV-tPA, the rate of hemorrhagic transformation remains fairly stable across studies. Even when administered in an early time window, IV-tPA is also associated with an increased risk of hemorrhagic complications. In the pooled analysis of the ATLANTIS, ECASS, and NINDS rt-PA stroke trials, hemorrhage was observed in 5.9% of the tPA treated patients and only 1.1% of patients in the control group [27]. When the treatment time window was extended to 4.5 h with the ECASS 3 trial, the hemorrhage rate did not increase, with treated patients having a rate of 2.4% compared with a rate of 0.2% in the placebo group [2]. Pooled analysis of tPA treatment trials up to 6 h found a hemorrhage rate of 5.9% vs. 1.1% in placebo and further concluded that there was no association between time and risk of bleeding [27]. This lack of temporal relationship was again seen in a subsequent meta-analysis [28]. The IST 3 trial which treated patients up to 6 h had a similar hemorrhage rate to the prior trials at 7%. And most recently, a meta-analysis of patients treated out to 9 h based on multimodal imaging found a symptomatic hemorrhage rate of 5% [13]. These studies are consistent with the findings of the current study that BBB disruption within an ischemic lesion is not time dependent despite varying widely within the population.

This study has several limitations. Although the method for calculating BBB integrity is based on prior studies, the 20% threshold used in this study has not been independently replicated, and it is therefore difficult to calculate exactly how many patients would be safe to treat in the extended time window. Differences in MRI scanners or acquisition parameters may affect BBB calculations and may have contributed to the variation in measurements. BBB disruption itself may not reflect the entire risk of hemorrhage. For instance, hemorrhage that occurs remote from the acute ischemic lesion would not be accounted for with the method used in this study. This may potentially be addressed by using a whole brain approach to measuring BBB disruption. Although we included all patients in the initial review, the population included in the final analysis had to have had an MRI in the window being studied, which may have introduced a bias. This type of approach to stroke management requires readily available MRI, thus transfer to tertiary centers may be needed.

## Conclusions

Acute stroke patients presenting with ischemic lesions beyond current thrombolytic treatment time windows do not demonstrate degradation of the BBB in a time-dependent manner. BBB disruption varies substantially across such a population and a sizeable portion of these patients may be safe to treat with IV-tPA. Future studies should test the ability of BBB integrity to identify patients who would benefit from thrombolysis in a randomized control trial.

## Data Availability

The data and materials using in this study are subject to oversight by the NIH Office of Human Subjects Research Protections (OHSRP). Requests for access to the data would require data sharing agreement approved by the OHSRP.

## Abbreviations

1.5 T: 1.5 Tesla
3 T: 3 Tesla
ADC: Apparent diffusion coefficient
AIS: Acute ischemic stroke
BBB: Blood-brain barrier
BBPI: Blood-brain permeability imaging
DSC: Dynamic susceptibility contrast
FLAIR: Fluid attenuated inversion recovery
GRE: Gradient recall echo
HT: Hemorrhagic transformation
IV-tPA: Intravenous tissue plasminogen activator
LSN: Last seen normal
mm: Millimeter
MPD: Mean permeability derangement
MRA: Magnetic resonance angiography
MRI: Magnetic resonance imaging
msec: Millisecond
NIH: National Institutes of Health
NIHSS: National Institutes of Health Stroke Scale
OHSRP: Office of Human Subjects Research Protections
PH-2: Parenchymal hematoma type 2
PWI: Perfusion weighted imaging
ROI: Region of interest
s: Seconds
SD: Symptom discovery
TE: Echo time
TOF: Time-of-flight
TR: Time to repetition
TTP: Time-to-peak
